# Dr. John Mackintosh

**Published:** 1838-01

**Authors:** 


### OBITUARY.

### DR. JOHN MACKINTOSH, OF EDINBURGH.

On the 28th of October, at Edinburgh, Dr. John Mackintosh, long known as a popular and successful medical teacher in that city. Dr. Mackintosh was the second son of Captain Mackintosh, of the 4th regiment of Foot, and was born at sea during the return of his mother from America, whither she had accompanied his father on service. John Mackintosh was destined for the medical profession at a very early age, or rather of his own accord formed the resolution of studying medicine, and could distinctly trace his predilection for the healing art to his having fractured his leg when a child. During the confinement consequent on that accident, he was so impressed with the usefulness of surgery, that he determined to make it the business of his life. After passing through the usual routine of education necessary for the student of medicine, Mr. Mackintosh was apprenticed, in 1804, to Messrs. Bell, Wardrop, and

Russell, at that time in high repute in Edinburgh as general practitioners and operating surgeons, and who in a great measure monopolized the surgical practice in the Royal Infirmary. Having finished his studies in 1808, he was immediately afterwards appointed a medical officer in the Royal Artillery, and served abroad in that respectable corps, principally in the West Indies and South America, and in the army of occupation in France some years after the battle of Waterloo.

In 1821, Dr. Mackintosh commenced practice as a physician in Edinburgh; and, in 1822, published a work on Puerperal Fever, with the view of elucidating the true nature of the dangerous malady which had recently committed dreadful ravages in Edinburgh and Leith. In 1823, he began a course of lectures on Midwifery and the Diseases of Women and Children, and proved a successful teacher of that department of medical science. In 1825, in compliance with a numerously signed requisition from the medical students then in Edinburgh, he commenced lecturing on the Principles of Pathology and Practice of Physic; and, since 1829, has been one of our most successful and popular teachers.

Dr. Mackintosh's greatest work, that on the Principles of Pathology and Practice of Physic, was first published in 1828, and has been extremely popular among students and junior practitioners, both in this country and in America. We gave a pretty full account of it in our Fifth Number.

Dr. Mackintosh was an ardent cultivator of medical science. His zeal in the cause of pathology is proved by the extent and variety of his splendid museum; and, what is particularly worthy of remark, his pathological researches were generally prosecuted with the view of being made subservient to the improvement of the practice of medicine. His zeal in the cause of science, as well as his philanthropy, were strikingly shewn during the prevalence of cholera in Edinburgh; no one having made such strenuous exertions in attending the sick and in attempts to throw light on the nature of the disease.

It is impossible to speak too highly of Dr. Mackintosh's qualifications as a practical physician. Acute powers of observation, presence of mind, gentleness, combined with resolution in the sick-chamber; whilst, in the consulting room, candour and openness to conviction, from whatever quarter it came, were the characteristics of his liberal mind.

Dr. M. possessed in a high degree most of the essential qualities of a good and successful teacher, although these were intermixed with striking defects; which also accompanied him, in some degree, beyond the walls of his lecture-room. He was too boastful and confident in his own opinions, and did not hesitate rashly to disparage others from whom he differed; but, with all this, there was an earnestness of purpose, and even a prevailing tone of generosity of feeling, which had an excellent effect upon his pupils; and, if he was boastful, his deeds were always at hand to support him, although not exactly to the extent which he claimed. With a good deal of rashness and occasional lack of discretion in his general conduct, he combined great activity and acuteness; and it must be admitted that no one has appeared for many years in the medical school of Edinburgh who produced so salutary an effect in awakening and stimulating the zeal both of teachers and students. He inspired the latter with enthusiasm for their profession, and made them active thinkers, instead of passive recipients of lifeless precepts.

Dr. Mackintosh was an honorable and an excellent man, and most estimable in all the relations of life: he was a warm-hearted, generous, and faithful friend, and one of the best of husbands and fathers; but, owing to his ardent temperament, his superabundant self-esteem, and his want of prudent caution, he had the misfortune to excite considerable hostility against him among his medical brethren, and to alienate some of his intimate friends. It is but justice to Dr. Mackintosh, however, to state that most of the differences of this kind originated in misconception or misrepresentation, as it was not in his nature to injure even an enemy.

Dr. Mackintosh was married early in life to Miss Mackenzie, of Bayfield, Rosshire, who survives him, together with one daughter and four sons.*

* The principal part of the above notice is taken from an article in *The Scotsman* newspaper, of November 8, 1837.—Eds.

